# Unique Pulmonary Hypertensive Vascular Diseases Associated with Heart and Lung Developmental Defects

**DOI:** 10.3390/jcdd10080333

**Published:** 2023-08-03

**Authors:** Hidekazu Ishida, Jun Maeda, Keiko Uchida, Hiroyuki Yamagishi

**Affiliations:** 1 Department of Pediatrics, Osaka University Graduate School of Medicine, 2-2 Yamadaoka, Suita 565-0871, Osaka, Japan; hideishi@ped.med.osaka-u.ac.jp; 2 Department of Cardiology, Tokyo Metropolitan Children’s Medical Center, 2-8-29 Musashidai, Fuchu 183-8561, Tokyo, Japan; jun_maeda@tmhp.jp; 3 Department of Pediatrics, Keio University of Medicine, 35 Shinanomachi, Shinjuku-ku 160-8582, Tokyo, Japan; hyamag@keio.jp; 4 Keio University Health Center, 4-1-1 Hiyoshi, Kohoku-ku, Yokohama 223-8521, Kanagawa, Japan

**Keywords:** congenital heart disease, developmental disorders, Fontan circulation, genetic analysis, major aortopulmonary collateral arteries, pulmonary hypertension, tetralogy of Fallot

## Abstract

Although pediatric pulmonary hypertension (PH) shares features and mechanisms with adult PH, there are also some significant differences between the two conditions. Segmental PH is a unique pediatric subtype of PH with unclear and/or multifactorial pathophysiological mechanisms, and is often associated with complex congenital heart disease (CHD), pulmonary atresia with ventricular septal defect, and aortopulmonary collateral arteries. Some cases of complex CHD, associated with a single ventricle after Fontan operation, show pathological changes in the small peripheral pulmonary arteries and pulmonary vascular resistance similar to those observed in pulmonary arterial hypertension (PAH). This condition is termed as the pediatric pulmonary hypertensive vascular disease (PPHVD). Recent advances in genetics have identified the genes responsible for PAH associated with developmental defects of the heart and lungs, such as *TBX4* and *SOX17*. Targeted therapies for PAH have been developed; however, their effects on PH associated with developmental heart and lung defects remain to be established. Real-world data analyses on the anatomy, pathophysiology, genetics, and molecular biology of unique PPHVD cases associated with developmental defects of the heart and lungs, using nationwide and/or international registries, should be conducted in order to improve the treatments and prognosis of patients with these types of pediatric PH.

## 1. Introduction

Pulmonary arterial hypertension (PAH) is a progressive and lethal disorder. Its estimated incidence is 2.5–7.5 cases per million per year and its prevalence is 15–50 cases per million individuals, according to French [1] and Scottish registries [2]. Also, its estimated prevalence is 4.8–8.1 cases per million for pediatric-onset [3] and 5.6–25 cases per million for adult-onset disease [4]. PAH is defined as a mean pulmonary arterial pressure of >20 mmHg and pulmonary vascular resistance of ≥3 Wood Units (WU) [5]. Childhood PAH is frequently associated with congenital heart disease (CHD) resulting from developmental defects of the cardiovascular system, and belongs to group 1.4.4 of the sixth World Symposium of Pulmonary Hypertension (WSPH) classification (Table 1) [6]. Pulmonary hypertension (PH) has a negative impact on the natural course of CHD and worsens the clinical status and overall outcome. Patients with PAH or CHD are a heterogeneous population. Other than group 1.4.4 of PAH associated with CHD, group 5.4 in the 6th WSPH includes several anomalies involving differential pulmonary blood flow under the category of “segmental PH”, indicating the distinct nature of these entities as compared with other forms of PH [7]. Some types of complex CHDs are associated with congenital abnormalities in the pulmonary vascular structure, leading to segmental PH [6]. Other complex CHDs are associated with a single ventricle after Fontan operation [7]. Although patients with Fontan circulation do not have PH, those with poor pulmonary circulation show similar pathological changes in the small peripheral pulmonary arteries, as well as pulmonary vascular resistance (PVR). This condition is termed pediatric pulmonary hypertensive vascular disease (PPHVD). Finally, recent advances in genetic analyses have identified genes responsible for PAH, which are implicated not only in pulmonary endothelial function, but also in the development of the heart and lungs. For instance, pathogenic variants of *TBX4* are strongly associated with developmental disorders of the lungs [7], while variants of *SOX17* are strongly associated with simple CHD.

In this review, we focus on unique pulmonary hypertensive vascular diseases associated with developmental defects of the heart and lungs, including segmental PH and PPHVD in the Fontan circulation, and the developmental genes *TBX4* and *SOX17.* Recent advances highlighting the pathophysiological mechanisms, clinical management, and genetic origins of these conditions are discussed.

## 2. Segmental PH Associated with Complex CHD

### 2.1. Overview of Segmental PH

Segmental PH is a unique subtype of pediatric PH with unclear and multifactorial underlying pathophysiological mechanisms [8]. The concept of segmental PH was introduced in the 2015 European Society of Cardiology (ESC)/European Respiratory Society (ERS) Guidelines for the Diagnosis and Treatment of PH, reflecting the 2013 discussions in the 5th WSPH [8]. In 2018, the 6th WSPH revised their recommendations based on updated research [6], with the Pediatric Task Force of the WSPH refining the definition, classification, epidemiology, diagnostics, and treatment of pediatric PH [7]. Although the latest ESC/ERC PH guidelines have revised the major clinical classification of segmental PH [6], it is still emphasized in the pediatric field and is classified in group 5.4 in the 6th WSPH as pediatric PH with complex CHD. These complex CHD types commonly include tetralogy of Fallot (TOF) with aortopulmonary collateral arteries (MAPCAs), and rarely include isolated pulmonary artery of ductal origin, absent pulmonary artery, and hemitruncus with unbalanced pulmonary circulation, etc. [7]. Segmental PH indicates PH affecting one or several lobes of the lungs to different extents, with the remaining lobes being normotensive [9].

### 2.2. Segmental PH Associated with MAPCAs

**Figure 1:**
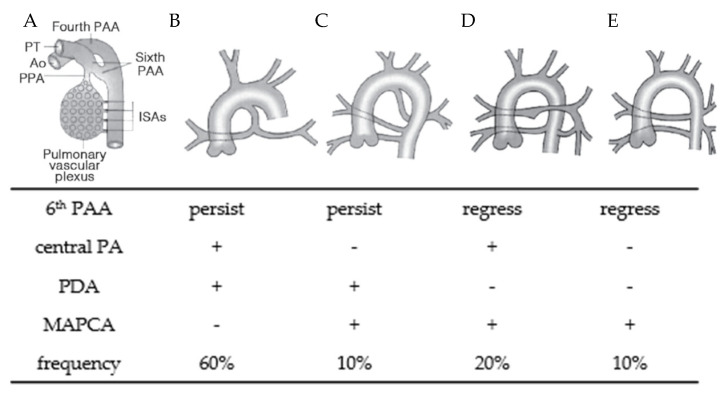
Development of the normal pulmonary artery and types of MAPCA [10]. (**A**) The developing pulmonary vascular system in the early embryonic stage. (**B**–**E**) Development of the pulmonary artery and MAPCA. The development of the sixth pharyngeal arch artery, pulmonary blood supply, and frequency of each type are shown in the table below the figure. Ao, aorta; ISA, intersegmental artery; MAPCA, major aorto-pulmonary collateral arteries; PAA, pharyngeal arch artery; PPA, peripheral pulmonary artery; PT, pulmonary trunk.

MAPCAs are developmentally aberrant vessels derived from the embryonic intersegmental artery. During the early fetal period, the vascular plexus in the lung buds is connected to the intersegmental arteries arising from the dorsal aorta (Figure 1A) [10,11]. This remains until fetal day 50, when the segmental arteries regress and the sixth pharyngeal arch gives rise to a central pulmonary artery that is connected to the right ventricle. Developmental defects in the sixth pharyngeal arch artery result in a diminutive central pulmonary artery and failure to develop the right ventricular outflow tract. Under these circumstances, the intersegmental arteries maintain the aorto-pulmonary connection and ensure pulmonary blood flow from the aorta after birth. The more severe the developmental defects of the sixth pharyngeal arch artery are, the more diminutive the central pulmonary artery becomes, with the development of MAPCAs (Figure 1B–E).

MAPCAs are commonly associated with TOF, particularly when atresia or severe stenosis of the pulmonary outflow tract exists. Clinically significant MAPCAs generally exceed 3 mm in diameter. As mentioned previously, MAPCAs are believed to originate from the intersegmental arteries connected to the splanchnic vascular plexus. After birth, MAPCAs mostly branch from the descending aorta but can also branch from the ascending aorta, subclavian artery, brachiocephalic artery, and—rarely—from the coronary artery [10,12], connecting with the vascular beds of the lungs. Morphologically, MAPCAs resemble dilated bronchial arteries and may be considered as a substitute for the physiological role of the pulmonary artery in terms of growth and vasoreactivity [12,13]. A histological study demonstrated the same histological findings as for the systemic arteries, with muscular media and distal merging into efficient pulmonary vascular beds [14]. However, the origins and characteristics of MAPCAs remain unclear [12,13,15].

**Figure 2:**
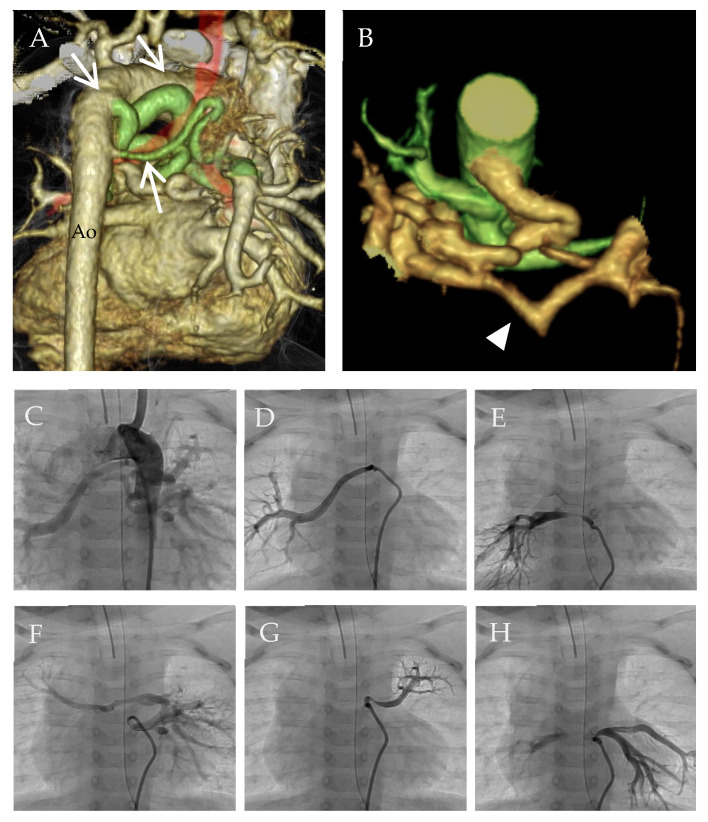
MDCT imaging and aortogram of MAPCA. (**A**) MAPCA (arrows) viewed from the back. (**B**) The central PA (arrowhead) and MAPCA. (**C**) Aortogram showing the total image of MAPCA. (**D**–**H**) Selective angiograms of MAPCA. Ao, descending aorta; MAPCA, major aorto-pulmonary collateral arteries; MDCT, multidetector computed tomography; PA, pulmonary artery.

In cases with MAPCAs, pulmonary overcirculation and undercirculation may coexist in different lung segments of the same patient [10]. When MAPCAs are non-stenotic, pulmonary vascular beds are prone to high shear stress under systemic arterial pressure. Excessive pulmonary blood flow may cause PH. On the other hand, peripheral stenosis in MAPCAs may protect some, but not all, pulmonary vascular beds against systemic blood pressure. Moreover, peripheral stenosis of MAPCAs may lead to the development of proximal PH, if severe, and may decrease pulmonary blood flow segmentally. This undercirculation may lead to hypoplasia of the pulmonary vascular beds in some lung segments, eventually increasing the vascular resistance and resulting in PH in corresponding segments. These changes can occur heterogeneously in the lungs of individuals with MAPCAs (Figure 2).

### 2.3. Surgical Treatment for MAPCAs to Avoid Segmental PH

Surgical intervention is an effective treatment option for creating an integrated unified pulmonary blood supply. Traditionally, multistage unifocalization of the MAPCAs or pulmonary rehabilitation has been performed. Unifocalization includes anastomosing the ipsilateral MAPCAs to unify them and then incorporating them into the central pulmonary artery, if this is present. Systemic pulmonary shunts are also used to maintain the pulmonary blood flow. Pulmonary rehabilitation involves promoting the growth of the hypoplastic central pulmonary artery by using a central aortopulmonary shunt or a right ventricle-to-pulmonary artery connection. Single-stage unifocalization has become the preferred surgical option as it can achieve a lower right ventricular pressure, a lower re-intervention rate, and is a simpler surgical procedure than multi-stage unifocalization and pulmonary rehabilitation [13,15]. After these palliative procedures, if the pulmonary vascular beds are well developed, segmental PH may not occur.

**Figure 3:**
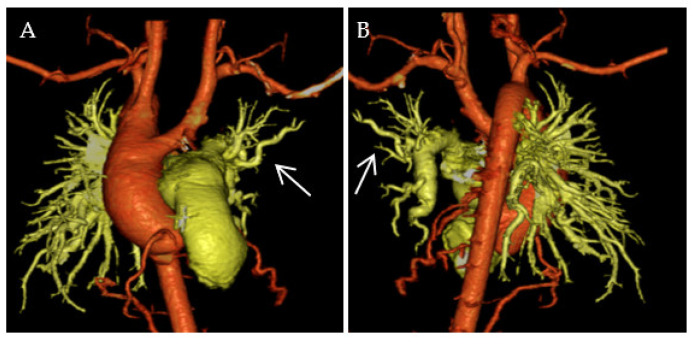
MDCT imaging of repaired TOF and MAPCA in frontal (**A**) and back (**B**) views. The left pulmonary arteries are hypoplastic and tortuous (arrows). MAPCA, major aorto-pulmonary collateral arteries; MDCT, multidetector computed tomography; TOF, tetralogy of Fallot.

Despite completing the surgical repair of TOF and MAPCAs, segmental PH may persist or may even develop [9]. It can be caused by hypoplasia, obstruction, or hypoxic spasm of the pulmonary vessels, in addition to original unrepaired conditions. Figure 3 shows a three-dimensional multidetector computed tomography imaging of an infant with repaired TOF, MAPCAs, and residual PH. The left pulmonary arteries were more hypoplastic and tortuous than the right pulmonary arteries, suggesting abnormal arborization. In such cases, surgical or catheter interventions, including angioplasty and stenting, may be indicated and medical treatments should be considered.

### 2.4. Pulmonary Vasodilators for Segmental PH

Pulmonary vasodilators are a medical option for targeting segmental PH. Although the exact origin of MAPCAs is not well understood, distal lesions of MAPCAs appear to be similar to those of pulmonary vascular architecture, as previously described, which supports the efficacy of pulmonary vasodilators [14]. Yasuhara and Yamagishi detailed the case of a 5-year-old girl with repaired TOF and MAPCAs who had segmental PH. They applied combination therapy using both percutaneous pulmonary angioplasty and pulmonary vasodilators, including a phosphodiesterase type 5 (PDE5) inhibitor, sildenafil, and the endothelin-receptor antagonist (ERA), bosentan, which successfully improved segmental PH [11]. Yamamura et al. reported the efficacy of bosentan for treating segmental PH in two pediatric patients with repaired TOF and MAPCAs [16]. Moreover, a few small-scale studies have described the effects of bosentan and/or sildenafil in patients with repaired TOF and MAPCAs with PH (Table 2). All study patients experienced remission of clinical symptoms [17,18,19], but their PH did not always improve, which is consistent with previous findings [9]. Some patients experienced adverse effects from pulmonary vasodilators, such as headache, nausea, lethargy, and dyspnea, including a patient requiring cessation of sildenafil treatment [17,18]. Further pulmonary vasodilators may cause pulmonary edema in sections of the lung where arterial pressures are normal, particularly in patients with diastolic dysfunction. Careful monitoring of patients after administration of these agents is needed.

Because the development and distribution of MAPCA are significantly heterogeneous and individually different, a patient-based approach is necessary to evaluate segmental PH to plan a treatment strategy. Pulmonary vasodilators appear to be effective in only a small number of patients with segmental PH; however, evidence is insufficient. The International Registry of PH associated with CHD is currently enrolling more than 700 patients [20] and is expected to provide a comprehensive overview of their management. In Japan, a nationwide registry of the Japanese Association of CHD-PH Registry (JACPHR) is also ongoing, and several patients with TOF and MAPCAs with segmental PH have been enrolled [21]. Accumulating clinical data on segmental PH may help delineate the most appropriate treatment strategy in future.

## 3. Pulmonary Hypertensive Vascular Disease in the Fontan Circulation

### 3.1. Overview of Fontan Circulation

In complex CHD with single-ventricle physiology, the Fontan procedure is a well-established palliative procedure. The original Fontan procedure was reported more than 50 years ago [22] and has been a landmark contribution for improving mortality and morbidity in patients with a single ventricle [23,24,25]. In Fontan circulation, the superior and inferior vena cava are directly connected to the pulmonary arteries without the subpulmonary ventricle. Pulmonary circulation after the Fontan procedure is unique, differing from that associated with biventricular physiology. The diastolic kinetics of the ventricle and central venous pressure (CVP) drive the pulmonary blood flow. Therefore, well-developed pulmonary vascular beds and low PVR are mandatory for establishing Fontan circulation [26,27]. Additionally, ventricular diastolic function and atrioventricular valve regurgitation, which contribute to high pulmonary wedge pressure, are additional factors for optimal Fontan circulation [28,29]. Most of the serious complications of Fontan patients are attributed to the high PVR and CVP [27], and in some cases of so-called “failing Fontan,” the pulmonary circulation is not fully functional. Chronic liver congestion with a high CVP is thought to induce liver fibrosis and inflammation, leading to cirrhosis and eventually to hepatic carcinoma, which is called Fontan-associated liver disease [30,31]. Chronic congestion of the intestines, kidneys, and lymphoid system can cause protein-losing enteropathy (PLE), plastic bronchitis, and renal failure [32,33]. These complications can increase overall mortality, and deteriorate exercise capacity and quality of life in Fontan patients [23,34]. Therefore, understanding the pulmonary vasculature biology and the effects of pulmonary vasodilators is important for managing Fontan patients.

### 3.2. Pulmonary Vascular Remodeling in Fontan Circulation

Several hypotheses have been proposed regarding the progression of pulmonary vascular remodeling in patients with Fontan disease. The most common pathogenic hypothesis is the lack of pulsatile blood flow in the pulmonary arteries of the Fontan circulation. Several previous studies, including animal models of nonpulsatile cavopulmonary anastomosis, have demonstrated that vascular endothelial function is significantly impaired under conditions of nonpulsatile pulmonary blood flow [35,36,37,38]. Pulsatile blood flow evokes optimal shear stress in endothelial cells and induces the expression of endothelial nitric oxide synthetase (eNOS), which induces NO production for vasodilatation. Impairment of eNOS production leads to vasoconstriction of the pulmonary arteries.

Another hypothesis involves endothelin (ET)-1, a potent vasoconstrictive protein. ET-1 has mitogenic activity in vascular smooth muscle cells and promotes vascular remodeling. The pathogenicity of ET-1 has been previously demonstrated for various types of PAH [39]. Previous clinical studies have demonstrated that plasma ET-1 levels are significantly elevated in Fontan patients and correlate positively with CVP [40,41]. Immunohistological and quantitative real-time polymerase chain reaction analyses demonstrated that the expression of not only ET-1 but also ET receptor types A and B was significantly elevated in the pulmonary arteries of failing Fontan patients as compared to those of healthy controls and non-failing Fontan patients [42]. In patients with failed Fontan circulation, the medial walls of the pulmonary arteries were thickened, and the intima was significantly hypertrophied. Histological findings of pulmonary arterial remodeling were similar to those of patients with idiopathic PAH (IPAH) [43]. Notably, patients with non-failing Fontan circulation who suddenly died of ventricular arrhythmia or infection showed relatively milder remodeling of the pulmonary arteries and lower levels of ET-1 and its receptors than in failing Fontan circulation patients with PLE or plastic bronchitis [42,44]. Although these studies did not analyze the expression levels of eNOS in the pulmonary arteries of failed Fontan patients, they indicated that ET-1 and its signaling pathway may play important roles in the pathogenic mechanisms of pulmonary arterial remodeling in the Fontan circulation and provide a rationale for the use of ERA to treat failing Fontan patients.

Recently, hypoxia-inducible factor (HIF)-1 has been considered as a major player in the pathogenesis of pulmonary vascular remodeling in patients with PAH [45]. The pulmonary vasculature is hypoxic in Fontan patients. Low cardiac output and insufficient vascularization and oxygenation can induce tissue hypoxia, even without cyanosis, after the Fontan operation [46]. Patients with complex CHD often experience hypoxic tissue conditions before and after the Fontan procedure. HIF-1 induces the expression of several downstream factors, including ET-1 and vascular endothelial growth factor (VEGF). VEGF, a growth factor that induces angiogenesis, is elevated in patients with Fontan disease [47]. VEGF plays an important role in the pathogenesis of PAH and in the remodeling of the pulmonary arterial vasculature. Taken together, multiple pathogenic signaling cascades may be involved in the remodeling seen in Fontan patients. Pulmonary vasculature remodeling progresses more readily, particularly in failing Fontan patients, rather than in biventricular repaired patients.

### 3.3. Pulmonary Vasodilators for Failing Fontan

Numerous pulmonary vasodilative drugs have beneficial effects on exercise capacity, morbidity, and mortality in patients with IPAH and hereditary PAH (HPAH). Considering the pathophysiology of the pulmonary vasculature in failing Fontan patients, pulmonary vasodilators are theoretically likely to be beneficial. Since it was reported that inhaled NO elicits acute vasoreactivity late after the Fontan operation, the potential therapeutic effects of pulmonary vasodilators have been extensively investigated [48]. A randomized controlled trial (RCT) was the first to report that treatment with sildenafil was effective for increasing cardiac output and exercise capacity in Fontan patients [49]. However, a later study found no beneficial effects of sildenafil on Fontan circulation, including the peak VO_2_ [50]. More recently, in a retrospective cohort study (RC), it was reported that the routine use of sildenafil soon after a Fontan operation did not improve postoperative morbidity or the length of hospital stay [51]. Udenafil, a recently developed PDE5 inhibitor, was administered to Fontan patients in the FUEL trial and was found to improve multiple measures of exercise performance, but not peak VO_2_ [52]. Bosentan, a potent ERA, was also provided to Fontan patients in an RCT and demonstrated several beneficial effects. Although Schuuring et al. showed that 6 months of treatment with bosentan did not provide benefits for Fontan patients, Shang et al. demonstrated that bosentan reduced the incidence of pulmonary arteriovenous fistulae and protein-losing enteropathy while improving heart function [53,54]. Another small size RCT demonstrated that ambrisentan, a more specific antagonist of the ET receptor type A, could improve the exercise capacity of adult Fontan patients [55]. Illoprost, a synthetic analog of prostacyclin, was also investigated in a small RCT, which found it capable of improving peak oxygen pulse and peak VO_2_ in Fontan patients, appearing to be particularly beneficial in patients with impaired exercise function [56].

Recently, meta-analyses of the efficacy and safety of pulmonary vasodilators in Fontan patients were published by Wang et al. [57] and Li et al. [58]. Peak VO_2_ was significantly improved with pulmonary vasodilator treatment, whereas no significant changes were noted in the subgroup analysis of ERA and PDE5 inhibitors. However, quality of life, assessed using the SF-36 score, did not significantly change with treatment. Wang et al. reported that the New York Heart Association (NYHA) functional class and mean pulmonary arterial pressure were significantly improved in the pulmonary vasodilator group [57]. However, Li et al. concluded that these parameters did not differ significantly [58]. Both meta-analyses demonstrated that the overall mortality was not significantly different between the drug and control groups. Pulmonary vasodilators were deemed to be safe for administration to Fontan patients (Table 3).

Taken together, the beneficial effects of pulmonary vasodilators in patients with Fontan disease have not been universally established. However, in some Fontan patients, pulmonary vasodilators can improve exercise capacity and NYHA functional class, regardless of improvement in pulmonary hemodynamics. The positive effects of pulmonary vasodilators in Fontan patients may be attributed to a complex phenomenon rather than to a simple improvement in pulmonary hypertension. The discrepancy in the effect of pulmonary vasodilators in patients with Fontan circulation may be mainly due to the marked variation in the etiology and pathology of failing Fontan circulation among patients; additionally, the cause of Fontan failure is not only dependent on PVR. Ventricular systolic and diastolic functions, atrioventricular valve regurgitation, systemic arterial-pulmonary shunts, and arrhythmias affect the exercise capacity and mortality in Fontan patients. Large-scale RCTs are required to determine which subpopulations of Fontan patients are more likely to respond to pulmonary vasodilators. However, a registry of PAH associated with CHD will provide promising real-world data for further analysis of pulmonary vasodilators in Fontan patients. The analysis of the COMPERA registry, published in 2021, included only nine patients with pulmonary vascular disease and Fontan circulation; however, the data suggested that monotherapy with PDE5 inhibitors may be preferable in Fontan patients [20]. Large-scale comprehensive registry analyses, such as our JACPHR study, would uncover the role of pulmonary vasodilators in Fontan circulation.

## 4. Genetic Origins of PAH Associated with Developmental Defects of the Heart and Lungs

### 4.1. Overview of Genetics in PAH

In approximately 70–87% of HPAH and 12–20% of IPAH patients, a genetic cause was identified in PAH-associated genes [6,59]. Early genetic analyses indicated an autosomal dominant mode of inheritance. Recent genetic analyses of large cohorts using high-throughput next-generation sequencing have defined the frequency of individuals with deleterious variants in known PAH-associated genes and identified novel risk genes [60,61,62] of which 17 were listed as pathogenesis-associated genes in the 6th WSPH reported in 2018 [59]. However, known susceptibility variants are not completely penetrant. Many individuals carrying monogenic risk variants do not develop PAH, while a subset of patients have deleterious variants in more than one PAH-associated gene.

HPAH is most commonly caused by heterozygous pathogenic variants in the gene encoding a member of the bone morphogenetic protein (BMP) receptor family of transmembrane serine/threonine kinases (BMPR2) [63,64,65]. To date, more than 800 independent pathogenic *BMPR2* variants have been identified [59]. The inheritance of PAH is autosomal dominant, with incomplete penetrance. For *BMPR2* variants, penetrance has been estimated to be approximately 30% [59,66]. *BMPR2* mutation carriers have a younger mean age of onset of PAH and are less responsive to PAH targeted therapy than noncarriers [60,67,68]. Genes responsible for hereditary hemorrhagic telangiectasia (HHT; Osler–Weber–Rendu disease) are also identified as PAH-predisposing genes: activin A receptor type II-like kinase 1 (*ACVRL1*), endoglin (*ENG*), mothers against decapentaplegic, drosophila, homolog of 4 (*SMAD4*), and those belonging to the BMP/transforming growth factor-beta (TGF-β) super family [64]. *ACVRL1* and *ENG* variants contribute to approximately 0.8% of the PAH cases [60]. Patients with PAH harboring an *ACVRL1* mutation are characterized by a young median age of onset of 20 years and subsequently develop HHT [69]. The frequency of variants in the growth differentiation factor 2 gene (*GDF2*), which encodes the ligand of BMPR2/ACVRL1 (BMP9), was reported to be approximately 1% in the PAH cases in European cohorts, but was higher in Chinese patients (approximately 6.7%) [60,62,70]. The prevalence of variants in SMAD genes, which encode the downstream mediators of BMP signaling, was much lower.

Consequently, loss of or dysfunction in the finely tuned balance between BMP signaling and TGF-β signaling is considered to be the major molecular defect involved in the predisposition to and disease progression of PAH. Recently, some newly identified deleterious genetic variants in additional genes have been implicated in childhood-onset PAH, such as genes encoding the transcription factors TBX4 and SOX17. These findings have revised our understanding of the mechanism underlying PAH associated with developmental defects of the heart and lungs (Table 4).

### 4.2. TBX4 in Pediatric PAH

*TBX4*, which encodes T-box transcription factors, together with *TBX5,* is crucial for embryonic development of the limbs and lungs [73,74]. Heterozygous *TBX4* variants cause the small patella syndrome (also known as the ischiocoxopodopatellar syndrome [ICPPS; OMIM #147891]). *TBX4* variants have been confirmed to be a substantial cause of PAH, accounting for up to 8% of HPAH and IPAH cases, with or without ICPPS [75,76]. Enrichment of pathogenic *TBX4* variants has been observed in pediatric patients with PAH [77]. Zhu et al. reported that patients with HPAH harboring a *TBX4* variant had a 20-year younger age of onset than that of *BMPR2* mutation carriers [76]. *TBX4* pathogenic variants, or copy number variant deletions, have been identified in patients with developmental disorders of the lungs, including acinar dysplasia, congenital alveolar dysplasia, and alveolar growth abnormalities [78,79,80,81]. It has been reported that patients with TBX4-related PAH have a low diffusion capacity in the lungs and maldevelopment of the lung buds [82]. While early reports indicated that some PAH patients with *TBX4* variants showed a milder presentation, it has recently been recognized that the clinical phenotypes associated with *TBX4* disruption represent a broad spectrum, ranging from transient neonatal PH to severe progressive or biphasic PH, with or without developmental disorders [77].

TBX4 is expressed throughout the lung mesenchyme during the early stages of embryogenesis [83,84]. In animal studies, a reduction in TBX4 and TBX5 levels in mice led to a severe decrease in branching [84]. We recently identified three novel *TBX4* variants associated with PAH. In vitro functional analyses have revealed that TBX4 directly regulates the transcriptional activity of the fibroblast growth factor 10 gene (*FGF10*), whereas the identified *TBX4* variant proteins failed to activate *FGF10* transcription because of disruption of the nuclear localization signal or poor DNA-binding affinity. *FGF10* is directly regulated by TBX4, expressed in the lung mesenchyme, and is involved in the initial lung bud formation stages from the ventral foregut to the outgrowth of the main bronchi; thus, the TBX4-FGF10 pathway plays a key role in subsequent branching morphogenesis [84,85,86]. Ex vivo inhibition of *TBX4* resulted in insufficient lung morphogenesis and specific downregulation of *TIE2* and Kruppel-like factor 4 gene (*KLF4*) expression. Our study suggested that variants in *TBX4* may lead to PAH through insufficient lung morphogenesis by disrupting the TBX4-mediated direct regulation of FGF10 signaling and pulmonary vascular endothelial dysfunction involving PAH-related molecules [71]. In contrast, Cai et al. have reported that TBX4 positively regulates SMAD1/5 crosstalk via BMP signaling [87]. Taken together, the impaired function of TBX4 may result in PH through two underlying mechanisms: (1) congenital lung hypoplasia could be the cause of group 3.5 PH with the developmental lung disorders in group 3 PH due to lung disease and/or hypoxia, and (2) dysfunction of pulmonary vascular endothelial cells could be the usual cause of group 1.2 PH: HPAH in group 1.

### 4.3. SOX17 in PAH Associated with CHD

*SOX17* encodes a member of the conserved SRY-related high-mobility group (HMG) box gene family of transcription factors that is widely expressed during development. *SOX17* and *SOX18*, which belong to the subgroup of *SOXF* genes, are crucial during embryonic development, and play a role in processes such as vasculogenesis, angiogenesis, arterial specification, and vascular remodeling [88,89,90]. During embryogenesis, *SOX17* is selectively expressed in arterial endothelial cells [91]. Endothelium-specific inactivation of *Sox17* in murine embryos or postnatal retinas leads to impaired arterial specification, embryonic lethality, or arteriovenous malformations, respectively [92]. It has also been reported that *SOX17* is associated with intracranial aneurysms in genome-wide association studies, while the endothelial-specific knockout of *Sox17* induced intracranial aneurysms [93]. Furthermore, conditional knockout of *Sox17* in mesenchymal progenitor cells demonstrated that SOX17 is required for the normal formation of lung microvessels in utero [94].

Childhood forms of PAH are frequently associated with CHD, forming group 1.4.4 of the 6th WSPH [5]. In 2018, *SOX17* was identified as a PAH-associated gene via gene burden testing in an IPAH cohort [95]. This study showed that one nonsense mutation detected in a family may underlie early-onset PAH associated with atrial septal defects (ASD). Whole-exome sequencing in a cohort of 256 cases with CHD confirmed *SOX17* as a major cause of PAH associated with CHD. In addition, the striking identification of rare deleterious missense variants in 9 of the 13 pediatric cases with simple CHD (i.e., ASD, patent ductus arteriosus, or ventricular septal defect) was carried out. The precise pattern of *SOX17* expression is controlled by master transcription factors for cardiogenesis, such as GATA4, MEF2C, TBX5, and NKX2-5 [96]. Moreover, SOX17 inhibits WNT/ß-catenin signaling through direct protein interaction, while *NOTCH1* is a direct transcription target of SOX17 during embryonic arterial development [97]. These molecular mechanisms may be implicated in the impairment of SOX17 in PAH and in the development of heart defects. Other reports of childhood-onset PAH included four likely pathogenic variants in a cohort of 2572 PAH cases from a PAH biobank, a report of 12 families [60], and 128 IPAH or HPAH index cases from Japan, in which *SOX17* variants were identified in four patients, among whom three patients had ASD or patent foramen ovale [98]. Most patients had severe clinical PAH with systemic or super-systemic resting pulmonary arterial pressure and intravenous vasodilator treatment. Furthermore, severe PAH was observed in all patients carrying variants in the conserved HMG-box domain, which is essential for DNA binding [95].

## 5. Conclusions

In this review, we summarized the recent advances in the field of unique PPHVD associated with developmental defects of the heart and lungs, focusing on segmental PH associated with complex CHD, Fontan circulation with single-ventricle CHD, and mutated genes associated with PAH and cardiopulmonary development, namely *TBX4* and *SOX17*. Notably, these entities are not classified in major PAH group 1, but in group 5, indicating unclear and/or multifactorial mechanisms or overlap between groups 1 and 3 in the 6th WSPH, suggesting complex multifactorial mechanisms [5]; however, they are very important in the clinical practice of pediatric cardiology. Therefore, relatively little is known about the clinical picture of and therapeutic strategies for these entities, although recent advances in pulmonary vasodilators have improved the prognoses of patients with typical IPAH and HPAH. Further investigation of real-world data on the anatomy, pathophysiology, genetics, and molecular biology of these PPHVDs associated with developmental defects of the heart and lungs based on nationwide and/or international registries would provide new insights into the disease and facilitate an understanding of the prognosis and therapies for these unique types of PAH.

## Figures and Tables

**Table 1:** Clinical classification of PH ^1^.

Group 1	PAH
1.1	IPAH
1.2	HPAH
1.3	Drug- and toxin-induced PAH
1.4	APAH
1.4.1	PAH associated with connective tissue disease (APAH-CTD)
1.4.2	PAH associated with human immunodeficiency virus infection
1.4.3	PAH associated with portal hypertension
1.4.4	APAH-CHD
1.4.5	PAH associated with schistosomiasis
1.5	PAH long-term responders to calcium channel blockers
1.6	PAH with overt features of venous/capillaries (PVOD/PCH) involvement
1.7	PPHN syndrome
Group 2	PH due to left heart disease
2.1	PH due to heart failure with preserved left ventricular ejection fraction
2.2	PH due to heart failure with reduced left ventricular ejection fraction
2.3	Valvular heart disease
2.4	Congenital/acquired cardiovascular conditions leading to post-capillary PH
Group 3	PH due to lung diseases and/or hypoxia
3.1	Obstructive lung disease
3.2	Restrictive lung disease
3.3	Other lung disease with mixed restrictive/obstructive pattern
3.4	Hypoxia without lung disease
3.5	Developmental lung disorders
Group 4	PH due to pulmonary artery obstructions
4.1	Chronic thromboembolic pulmonary hypertension
4.2	Other pulmonary artery obstructions
Group 5	PH with unclear and/or multifactorial mechanisms
5.1	Hematological disorders
5.2	Systemic and metabolic disorders
5.3	Others
5.4	Complex CHD

^1^ Updated clinical classification according to the sixth World Symposium on Pulmonary Hypertension. Group 1 comprises subgroups of PAH, with IPAH (1.1), HPAH (1.2), and APAH-CHD (1.4.4) being most common in children. PH, pulmonary hypertension; PAH, pulmonary arterial hypertension; IPAH, idiopathic pulmonary arterial hypertension; HPAH, heritable pulmonary arterial hypertension; APAH, associated pulmonary arterial hypertension; CHD, congenital heart disease; PPHN, persistent pulmonary hypertension of the newborn.

**Table 2:** The effect of pulmonary vasodilators in cases with repaired pulmonary atresia with ventricular septal defect and major aortopulmonary collateral arteries with pulmonary hypertension.

Study	n	Age (Year) Median (Range)	Duration (Year) Median (Range)	Medication	Outcome
Lim et al. [17]	5	19 (17–47)	(0.5–2)	S: 5	Symptoms were improved in 5 cases. 6 MWT was increased in 2 cases.
Schuuring et al. [18]	7	32 (23–42)	1	B: 7	Symptoms were improved in 7 cases. 6 MWT was increased in 6 cases.
Apostolopoulou et al. [19]	3	26 (21–31)	10 (5–14)	B 3	Symptoms were improved in 3 cases.

B, bosentan; PVR, pulmonary vascular resistance; RVP, right ventricular pressure; S, sildenafil; 6 MWT, six-minute walking test.

**Table 3:** The effect of pulmonary vasodilators in pulmonary hypertensive vascular disease related to Fontan circulation.

Study	Year	Country	Design	Age	No. of Patients	Drugs	Outcomes	Conclusions
Giardini et al. [49]	2008	Italy	RCT	22.8 ± 4.9	27	Sildenafil 0.7 mg/kg	peak VO_2_	Sildenafil improved exercise capacity, pulmonary blood flow, and cardiac index
Goldberg et al. [50]	2011	USA	RCT	14.9 ± 5.1	55	Sildenafil 60 mg/day	peak VO_2_	Sildenafil did not improve VO_2_ max. However, increased ventilatory efficiency and improved oxygen consumption occurred in two subgroups
Collins et al. [51]	2017	USA	RC	4.0 ± 0.3	103	Sildenafil 1.05–3.0 mg/kg/day	Chest tube output Length of hospital stay Duration of mechanical ventilation	Routine early administration of sildenafil after Fontan operation did not improve postoperative chest tube output, length of stay, or duration of mechanical ventilation.
Goldberg et al. [52]	2020	USA	RCT	15.5 ± 2.0	400	Udenafil 175 mg/day	peak VO_2_ Anaerobic threshold Myocardial performance index BNP	Udenafil could not improve peak VO_2_, but improved multiple measures of exercise performance at the anaerobic threshold.
Schuuring et al. [53]	2013	Netherlands	RCT	28 (18–55)	42	Bosentan 250 mg/day	Exercise capacity NT-proBNP SF-36 quality of life NYHA class	Six months treatment of bosentan was not beneficial.
Shang et al. [54]	2013	China	RCT	2.5–18	39	Bosentan, 31.25–250 mg/day according to body weight	Mortality PLE PAF 6MWD NYHA class	Bosentan therapy in post-Fontan patients could reduce the incidence of PAF and PLE and improve heart function.
Cedars et al. [55]	2016	USA	RCT	24.9 ± 5.2	47	Ambrisentan 10 mg/day	peak VO_2_ SF-36 quality of life	Ambrisentan improved exercise capacity in adult Fontan patients.
Rhodes et al. [56]	2013	USA	RCT	median 16.7	18	Illoprost 5.0 μg	peak VO_2_	Iloprost improved the peak oxygen pulse and peak VO2 of Fontan patients and appeared to be particularly beneficial among patients with impaired exercise function.

**Table 4:** Genetic variants in pulmonary arterial hypertension (PAH) associated with developmental defects of the heart and lungs.

Gene	Pulmonary Hypertension Phenotypic Association	Putative Molecular Mechanism	Inheritance Pattern	Associated Clinical Features	Populations
*TBX4* [62,71,72]	Heritable and idiopathic PAH ischiocoxopodopatellar syndrome Parenchymal lung disease Bronchopulmonary dysplasia Persistent pulmonary hypertension of the neonate	Loss of function	Autosomal dominant	Patellar aplasia Skeletal abnormalities, particularly pelvis, knees, and feet	Pediatric and (less commonly) adult
*SOX17* [62]	Heritable and idiopathic PAH Congenital heart disease	Unknown	Autosomal dominant	Atrial septal defect, patent ductus arteriosus, and ventricular septal defect	Pediatric and adult

## Data Availability

Not applicable.
